# Physiology and Pathophysiology of Steroid Biosynthesis, Transport and Metabolism in the Human Placenta

**DOI:** 10.3389/fphar.2018.01027

**Published:** 2018-09-12

**Authors:** Waranya Chatuphonprasert, Kanokwan Jarukamjorn, Isabella Ellinger

**Affiliations:** ^1^Pathophysiology of the Placenta, Department of Pathophysiology and Allergy Research, Center for Pathophysiology, Infectiology and Immunology, Medical University of Vienna, Vienna, Austria; ^2^Faculty of Medicine, Mahasarakham University, Maha Sarakham, Thailand; ^3^Research Group for Pharmaceutical Activities of Natural Products Using Pharmaceutical Biotechnology (PANPB), Faculty of Pharmaceutical Sciences, Khon Kaen University, Khon Kaen, Thailand

**Keywords:** cholesterol, progestagens, estrogens, glucocorticoids, gestational diabetes mellitus, preeclampsia, intrauterine growth retardation, oxysterols

## Abstract

The steroid hormones progestagens, estrogens, androgens, and glucocorticoids as well as their precursor cholesterol are required for successful establishment and maintenance of pregnancy and proper development of the fetus. The human placenta forms at the interface of maternal and fetal circulation. It participates in biosynthesis and metabolism of steroids as well as their regulated exchange between maternal and fetal compartment. This review outlines the mechanisms of human placental handling of steroid compounds. Cholesterol is transported from mother to offspring involving lipoprotein receptors such as low-density lipoprotein receptor (LDLR) and scavenger receptor class B type I (SRB1) as well as ATP-binding cassette (ABC)-transporters, ABCA1 and ABCG1. Additionally, cholesterol is also a precursor for placental progesterone and estrogen synthesis. Hormone synthesis is predominantly performed by members of the cytochrome P-450 (CYP) enzyme family including CYP11A1 or CYP19A1 and hydroxysteroid dehydrogenases (HSDs) such as 3β-HSD and 17β-HSD. Placental estrogen synthesis requires delivery of sulfate-conjugated precursor molecules from fetal and maternal serum. Placental uptake of these precursors is mediated by members of the solute carrier (SLC) family including sodium-dependent organic anion transporter (SOAT), organic anion transporter 4 (OAT4), and organic anion transporting polypeptide 2B1 (OATP2B1). Maternal–fetal glucocorticoid transport has to be tightly regulated in order to ensure healthy fetal growth and development. For that purpose, the placenta expresses the enzymes 11β-HSD 1 and 2 as well as the transporter ABCB1. This article also summarizes the impact of diverse compounds and diseases on the expression level and activity of the involved transporters, receptors, and metabolizing enzymes and concludes that the regulatory mechanisms changing the physiological to a pathophysiological state are barely explored. The structure and the cellular composition of the human placental barrier are introduced. While steroid production, metabolism and transport in the placental syncytiotrophoblast have been explored for decades, few information is available for the role of placental-fetal endothelial cells in these processes. With regard to placental structure and function, significant differences exist between species. To further decipher physiologic pathways and their pathologic alterations in placental steroid handling, proper model systems are mandatory.

## Introduction

The placenta is a multifunctional organ enabling optimal fetal growth. Structure and function can adapt to diverse external stressors. In case of failure of adaptation or inadequate placental development, fetal survival or fetal growth and development are endangered and developmental programming of adult diseases may occur (Barker and Thornburg, 2013; Burton et al., 2016; Arabin and Baschat, 2017). Moreover, the placenta contributes to maternal diseases such as preeclampsia, which predispose the mother to lifelong illness (Bokslag et al., 2016).

Essential placental functions are biosynthesis, metabolism, and transport of cholesterol, sex hormones and glucocorticoids. This article summarizes placental handling of these steroids under physiologic conditions and gives an overview on changes observed due to maternal diseases and exogenous influences. Whenever possible, data obtained in human placenta or human *in vitro* systems are referenced. We also draw attention to open research questions.

## The Human Placental Barrier And Placental Model Systems

**FIGURE 1:**
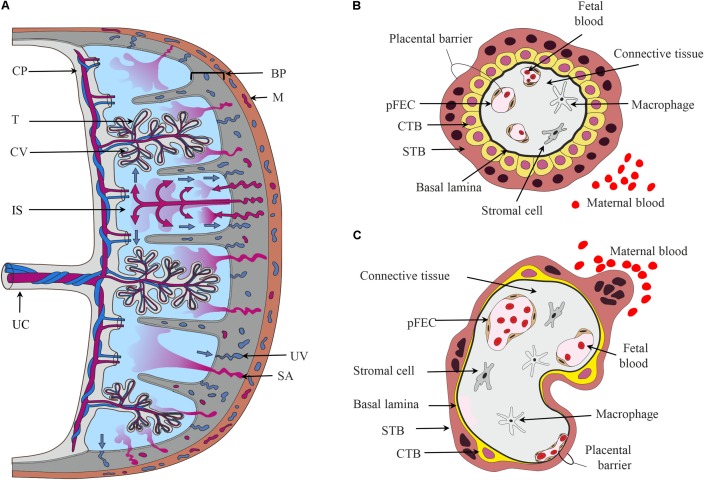
The placental barrier. **(A)** Schematic depiction of the main structural elements of the human placenta. From the chorionic plate (CP), the umbilical cord (UC), and the chorionic villi (CV) originate. The umbilical vein carries oxygen- and nutrient-rich blood from the placenta to the fetus, while two arteries transport deoxygenated blood and waste products from the fetus to the placenta. The intervillous space (IS) is filled with maternal blood that enters this cavity via remodeled and opened maternal spiral arteries (SA) and leaves via uterine veins (UV). Cells in direct contact with maternal blood are the villous trophoblasts (T). The basal plate (BP) contains extravillous trophoblasts and decidual cells. **(B,C)** Schematic representation of first trimester **(B)** and term trimester chorionic villi **(C)** depicting the major cell types and the placental barrier. CTB, cytotrophoblast; M, myometrium of the maternal uterus, pFECs, placental-fetal endothelial cells; STB, syncytiotrophoblast.

The hemochorial human placenta results from a deep invasion of embryonic cells during implantation. The mature human placenta is a disk delimited by chorionic and basal plate (**Figure 1A**), which enclose the intervillous space filled with maternal blood. The basal plate contacts the uterine wall. From the chorionic plate, both umbilical cord and the branched chorionic villi originate. The cells of the chorionic villi form the “placental barrier,” which prevents direct contact of maternal and fetal blood. Biosynthesis, metabolism, and transport of steroids occur in the chorionic villi (Benirschke et al., 2012).

Chorionic villi are composed of several cell types (Jones and Fox, 1991; Benirschke et al., 2012). The STB, an epithelial multinucleated syncytium, covers the surface of the chorionic villi. The microvillous apical membrane of the STB contacts maternal blood, while the basal plasma membrane of the STB is directed toward the stromal core of the villi. Apical and basal plasma membranes of the STB are often regarded as the most important membrane barriers in the materno-fetal transport processes. Nutrients, hormones, or fetal waste products traverse the STB by different transport mechanisms (Desforges and Sibley, 2010; Brett et al., 2014). Mononuclear CTB are located below the STB. They proliferate and fuse into the STB, thus supporting growth and regeneration of this layer. Early in pregnancy, CTBs form a continuous layer beneath the STB (**Figure 1B**). With progressing pregnancy, CTBs transform from a cuboidal into a flat phenotype. The CTB layer becomes incomplete, but maintains a functional network due to multiple interconnecting cell processes (Jones et al., 2008; **Figure 1C**).

The stromal core of the chorionic villi contains fetal blood vessels delineated by pFECs (Burton et al., 2009), macrophages (Reyes et al., 2017) and additional stromal cells (Sati et al., 2007), which are all embedded in a non-cellular matrix. pFECs are non-fenestrated endothelial cells. Early placental pFECs are probably more permeable than term pFECs. Recent years of research have demonstrated the importance of pFEC function for the fetal development (Wadsack et al., 2012). Heterogeneity of pFECs in the macro-circulation (umbilical cord) and microcirculation (chorionic villi) was shown *in situ* (Lang et al., 1993) and *in vitro* (Lang et al., 2003). Moreover, venous and arterial pFECs, which differ in their phenotypic, genotypic, and functional characteristics, have been described (Lang et al., 2008). The term placenta has a high degree of vascularity (Zhang et al., 2002). The capillaries closely approximate the villous covering, thereby forming “vasculosyncytial membranes” that are important for materno-fetal exchange processes (Burton et al., 2009). Thus, the term maternal–fetal interface or placental barrier consists of a thin cytoplasmic layer of STB apposed to a capillary (**Figure 1C**). Between the STB and pFEC the extracellular matrix is reduced to their fused basal laminae. STB and pFEC actively regulate uptake, metabolism, and transfer/exchange of molecules, while the non-cellular structures probably act as filters and provide transient storage capability (Benirschke et al., 2012).

The placenta undergoes significant anatomical changes in the course of pregnancy (Kingdom et al., 2000), which are relevant to maintain appropriate placental function as pregnancy progresses. Hormones in the fetal and maternal circulations have an important role in determining the placental phenotype (Fowden et al., 2015). As a developing organ that constantly adapts to the maternal environment, not only the structure, but also the transcriptome changes over time (Cox et al., 2015). In line with this, expression of various placental genes involved in biosynthesis, transfer or metabolism of steroids changes during pregnancy (see below).

The structure of the placenta is species-specific. Thus, no perfect animal model for the human placenta exists and special care must be taken with extrapolation of data from one species to another. Higher order primates including old world monkeys such as baboons are most closely aligned to humans with respect to structure as well as regulation of steroidogenesis (Pepe and Albrecht, 1995; Grigsby, 2016). Species-specific placental anatomy, endocrine function or advantages and limitations of relevant animal models to study the function of the human placenta in health and disease are detailed in various review articles (Malassine et al., 2003; Carter, 2007; Fowden et al., 2015; Carter and Enders, 2016; Grigsby, 2016; Hafez, 2017).

Placental functions can also be studied in human *ex vivo* models such as the isolated perfused placenta and placental villous tissue explants. Commonly used *in vitro* models are (1) isolated and *in vitro* cultured placental primary cells including trophoblasts as well as primary arterial and venous pFECs; (2) diverse, mainly trophoblastic cell lines derived by either transfection or spontaneous mutation including the choriocarcinoma cell lines BeWo, JEG-3, and Jar; and (3) isolated membrane vesicles (Lang et al., 2008; Prouillac and Lecoeur, 2010; Orendi et al., 2011; Cvitic et al., 2013; Myllynen and Vahakangas, 2013; Gohner et al., 2014; Steinberg and Robins, 2016). Choriocarcinoma cell lines are the most extensively used cell models. Their disadvantage is their origin from tumors. GeneChip analysis has revealed considerable differences between the gene expression patterns of choriocarcinoma cell lines and primary placental cells (Bilban et al., 2010). Thus, results obtained in cell lines should always be interpreted carefully and best be confirmed in other *in vitro* or *ex vivo* models. Over the last years co-cultures of trophoblast cells with endothelial cells have been established in order to mimic the entire placental barrier (Levkovitz et al., 2013a,b; Blundell et al., 2016, 2018). In the future, they may become attractive models to study transplacental transport processes and functional interdependence of cells.

## Cholesterol

**FIGURE 2:**
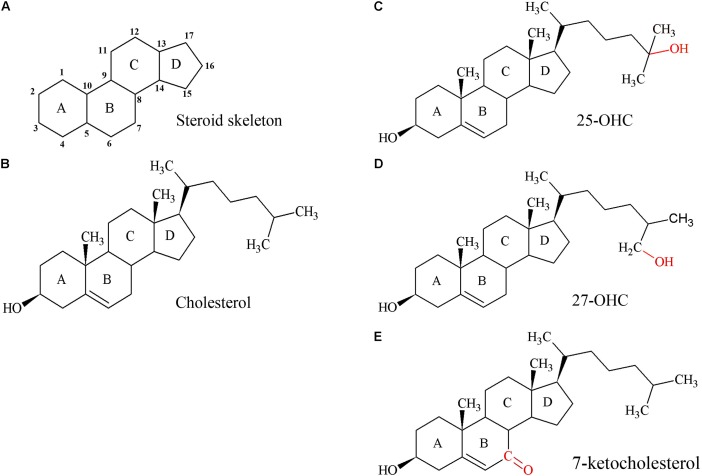
Structures of the steroid skeleton, cholesterol, and common oxysterols. **(A)** All steroids have the same basic perhydro-1,2-cyclopentenophenanthrene skeleton. Letters designate each ring, the carbon atoms are numbered. A slight variation in this skeleton or the introduction of functional groups result in various classes of steroids. **(B)** Unesterified cholesterol contains this skeleton with a hydroxyl group, two methyl groups, and a hydrogen tail. In the esterified form, a fatty acid would be bound to the hydroxyl group by an ester bond. **(C)** 25-hydroxycholesterol (25-OHC), the most extensively studied oxysterol. **(D)** Oxysterol 27-hydroxycholesterol (27-OHC). **(E)** Oxysterol 7-ketocholesterol. Red molecules indicate the positions of hydroxylation **(C,D)** or oxidation **(E)** of cholesterol to 25- or 27-OHC, or 7-ketocholesterol, respectively. Oxysterols are intermediates of cholesterol catabolism and act as signaling molecules with regulatory impact on various cellular processes including lipid metabolism.

Cholesterol (**Figure 2B**) is an essential component of cell membranes influencing their integrity, fluidity, and permeability, equally important for the growing placenta and fetus. The human placenta needs more than 1 g of cholesterol for tissue growth (Pratt et al., 1946) and the term human placenta manufactures approximately 400 mg of sex steroids from the precursor cholesterol per day (Knopp et al., 1981). Cholesterol is essential for myelination (Snipes and Suter, 1997) and as an activator and propagator of the sonic hedgehog-signaling pathway (Blassberg and Jacob, 2017). Thus, cholesterol is indispensable for patterning and development of the fetal nervous system.

Oxysterols are structurally closely related to cholesterol (**Figures 2C–E**) and have regulatory functions in cholesterol metabolism (Schroepfer, 2000; Mutemberezi et al., 2016; Sun et al., 2018). They are not only generated by enzymes, but also formed by autoxidation and thus accumulate under increased oxidative stress (Zarrouk et al., 2014).

According to the WHO, in 2008 the global prevalence of raised total cholesterol among female adults (≥5.0 mmol/l) was 40%; thus, hypercholesterolemia became a major health care problem. Various studies have indicated that maternal hypercholesterolemia (Napoli et al., 1997, 1999; Marceau et al., 2005; Catov et al., 2007; Zhang et al., 2017), but also maternal hypocholesterolemia (Sattar et al., 1999; Edison et al., 2007) negatively impact pregnancy outcome. Among the observed consequences are preterm delivery (Marceau et al., 2005; Catov et al., 2007; Edison et al., 2007), low birth weight (Marceau et al., 2005), IUGR (Sattar et al., 1999), and changes in the fetal aorta that determine the long-term susceptibility of children to fatty-streak formation and subsequent atherosclerosis (Napoli et al., 1997, 1999). Furthermore, altered mRNA expression levels of placental lipoprotein receptors involved in cholesterol uptake were observed (Ethier-Chiasson et al., 2007; Zhang et al., 2017).

As described below, also diseases of pregnancy and various endogenous and exogenous compounds can affect the placental proteins involved in cholesterol biosynthesis, metabolism, and transport. The long-term consequences for the fetus are hardly known. Thus, we need to further explore the regulation of cholesterol-associated pathways in placentas in healthy and diseased pregnancies in order to understand the correlation between observed changes *in utero* and diseases developing during later lives.

### Cholesterol Biosynthesis and Homeostasis

**FIGURE 3:**
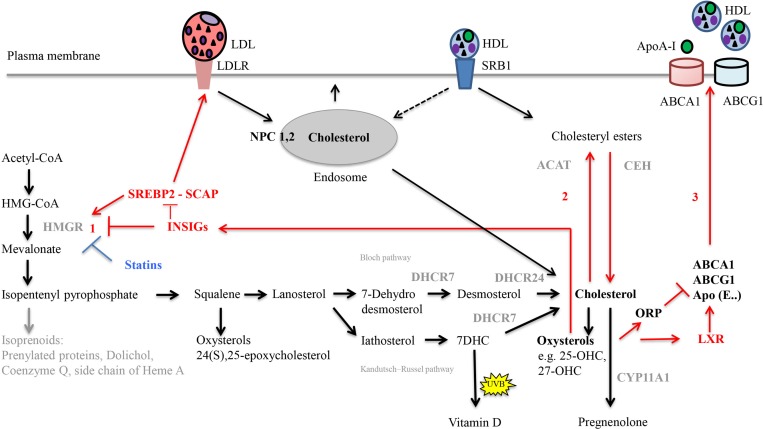
Important steps in cholesterol synthesis and homeostasis. Cholesterol synthesis occurs via the mevalonate pathway and involves over 20 enzymes. It starts from acetyl-CoA in the cytosol, but all steps downstream of 3-hydroxy-3-methylglutaryl-coenzyme A (HMG-CoA) occur in the smooth endoplasmic reticulum. Lanosterol, the first sterol, feeds either into the Bloch pathway or the Kandutsch–Russell pathway. In these pathways, cholesterol is produced from either desmosterol or 7-dehydrocholesterol (7-DHC), by the enzymes 24-dehydrocholesterol reductase (DHCR24) and 7-dehydrocholesterol reductase (DHCR7), respectively. Gene mutations of the enzyme DHCR7 result in Smith-Lemli-Opitz syndrome, SLOS, with increased levels of cholesterol precursors and reduced levels of cholesterol. In the skin, 7-DHC can be converted to vitamin D by UVB light. Diverse oxysterols are produced enzymatically from cholesterol, its precursors but also during subsequent steroid hormone synthesis. The rate limiting step in cholesterol synthesis is catalyzed by 3 HMG-CoA reductase (HMGR). Cellular homeostasis of cholesterol is maintained by three distinct mechanisms (red arrows). (1) Regulation of HMGR activity and levels to control cholesterol biosynthesis occurs by feed-back inhibition, control of gene expression, rate of enzyme degradation, and phosphorylation–dephosphorylation. For example, the transcription factor sterol regulatory element-binding protein 2 (SREBP2) positively regulates the gene expression of HMGR. Rising cholesterol and oxysterol levels reduce the rate of cholesterol biosynthesis by modulating the activities of insulin-induced gene (INSIG) proteins. When activated, INSIG both promotes the ubiquitination and consequent destabilization of HMGR and inhibits the transcriptional activity of SREBP2 by retaining it in complex with SREBP cleavage-activating protein (SCAP) in the endoplasmic reticulum. (2) Rising cholesterol levels also activate acyl-coenzyme A:cholesterol acyltransferase (ACAT), which esterifies cholesterol leading to its sequestration in cytosolic lipid droplets. Through hydrolysis via the cholesteryl ester hydrolase (CEH) enzyme system, the cholesteryl esters can be reused later. (3) Regulation of cholesterol uptake and export via low-density lipoprotein (LDL)-receptor-mediated uptake and high-density lipoprotein HDL-mediated reverse transport, respectively. Oxysterols activate liver-X receptor (LXR) transcription factors, which positively regulate the transcription of proteins that drive cholesterol efflux from the cell (ABC transporter, ABCA1 and ABCG1), and sequester it in lipoprotein particles containing Apolipoprotein E (ApoE) in the circulatory system. Activation of oxysterol binding protein-related proteins (ORP) by oxysterols negatively regulates cholesterol efflux by promoting ABCA1 ubiquitination and degradation. Following binding of lipoprotein particles (LDL and HDL) to their respective receptors [LDLR, Scavenger receptor class B type 1 (SRB1)], they are internalized into endosomes. Alternatively, HDL particles can transfer cholesteryl esters to the plasma membrane (selective lipid uptake) without requirement for endocytosis. Within endosomes, Niemann-Pick C1 (NPC1) and NPC2 are critical for the egress of internalized cholesterol from endosomes; they act together to redistribute cholesterol to the ER. Statins are HMGR inhibitors. The net result of statin treatment is an increased cellular uptake of LDLs, since the intracellular synthesis of cholesterol is inhibited and cells are therefore dependent on extracellular sources of cholesterol.

Cellular cholesterol homeostasis includes tightly regulated processes, which are summarized in **Figure 3** (Ikonen, 2006; Cerqueira et al., 2016; Soffientini and Graham, 2016). Intracellular cholesterol synthesis starts from acetyl-coenzyme A (acetyl-CoA). Lanosterol, the first sterol, feeds into two pathways, the Bloch (Bloch, 1965) and the Kandutsch–Russell (Kandutsch and Russell, 1960) pathway that both result in cholesterol production. The rate-limiting and committed step is the conversion of HMG-CoA to mevalonate mediated by HMGR. HMGR is regulated by endogenous molecules including transcription factor SREBP2 (Shimano and Sato, 2017) as well as the statin drugs (Lamon-Fava, 2013). Excess cellular cholesterol gets fatty-acylated by the action of ACAT, to form cholesteryl esters for storage in cytoplasmic lipid droplets; the inverse reaction is controlled by, e.g., CEH (Miller and Bose, 2011; Korber et al., 2017).

Cholesterol synthesis and HMGR activity in human (Hellig et al., 1970; Telegdy et al., 1970; Boguslawski and Sokolowski, 1984) as well as baboon placenta (Khamsi et al., 1972; Shi et al., 1999) decrease as pregnancy progresses. There are estimates that *de novo* cholesterol synthesis in the term human placenta provides only 1–2% of the cholesterol required for progesterone biosynthesis (Simpson et al., 1978). But the extent of cholesterol synthesis is species-specific; rabbit placentas near term exhibit a high level of HMGR expression and activity (Montoudis et al., 2003; Marseille-Tremblay et al., 2007).

Human pregnancy is characterized by maternal hyperlipidemia especially during the last trimester (Herrera et al., 2006). High maternal estrogen concentrations and maternal insulin-resistance stimulate hepatic VLDL production. Triglyceride and cholesterol concentrations in LDL and HDL particles rise, providing ample cholesterol fuel for the STB (Winkler et al., 2000; Herrera, 2002; Weissgerber and Wolfe, 2006). In contrast, maternal serum cholesterol levels in pregnant rabbits drop significantly compared to the non-pregnant state (Marseille-Tremblay et al., 2007).

With advancing gestational age, maternal serum-derived cholesterol replaces endogenously produced cholesterol as the major substrate of placental progesterone production in humans and baboons (Baird et al., 1973; Babischkin et al., 1997a,b; Henson, 1998; Shi et al., 1999). Addition of LDL to primary trophoblast cultures drastically suppresses *de novo* cholesterol synthesis, stimulates progesterone production and inhibits cholesteryl ester-forming ACAT (Winkel et al., 1980a,b, 1981). Likewise, addition of HDL_2_ stimulates progesterone secretion *in vitro* (Lasuncion et al., 1991). Trophoblasts isolated from healthy early, mid, and late baboon gestation show an upregulation of LDLR in mid and late gestation, while HMGR activity is reduced (Henson et al., 1997). Nevertheless, when external LDL supply is reduced *in vivo* or *in vitro*, human and baboon trophoblasts continue to produce sufficient progesterone due to endogenous cholesterol production (Simpson et al., 1978; Moise et al., 1986; Parker et al., 1986; Henson et al., 1991). In choriocarcinoma cells, an inverse relationship between the lipoproteins in the culture medium and the HMGR activity was demonstrated (Simpson et al., 1978). Together, these data suggest feedback inhibition of maternal-derived cholesterol on endogenous cholesterol synthesis and cholesteryl ester formation in human and baboon STB.

### Impact of Exogenous and Endogenous Factors on Placental Cholesterol Biosynthesis

Inconsistent with feedback inhibition by cholesterol, maternal hypercholesterolemia does neither change placental HMGR protein levels nor change placental cholesterol or cholesteryl ester content (Marseille-Tremblay et al., 2008).

From other tissues, age- and gender-related dysregulation of cholesterol metabolism, and specifically of HMGR regulation are known (Trapani and Pallottini, 2010). A hint for placenta-specific regulation of cholesterol biosynthesis is the observation that maternal hypercholesterolemia causes an increase in the placental expression of the transcription factor SREBP-2 (Marseille-Tremblay et al., 2008). Scarce information is available on regulation of the placental SREBP – SCAP – INSIG system (**Figure 3**). In the Golden Syrian hamster, suppression of sterol synthesis by exogenous sterol is blunted in placenta and other developing tissues when compared to parental tissues. This lack of response appears to be mediated at least partly through the SCAP:INSIGs ratio, which is 1.8-fold greater in the placenta as compared to the adult liver (Yao et al., 2007). Neither in human nor baboon placenta regulation of these molecules has been investigated so far, but this topic might be of relevance in the context of ART, which are increasingly applied today. Among the observed adverse neonatal outcomes in pregnancies conceived through *in vitro* fertilization and intracytoplasmic sperm injection are low birth weight and small size for gestational age. Interestingly, a recent study performed in humans (Lou et al., 2014) found that placenta and fetus from ART pregnancies showed altered transcript levels of *INSIG1* and *SREBF1*. The enhanced gene expression correlated with lower methylation rates of INSIG1 and SREBF1. The authors hypothesized an impact of ART on the placental/fetal cholesterol metabolism with consequences for the future life of the offspring (Collier et al., 2009). More research is required to confirm this theory.

Estrogen production is significantly (3–8 times at term) increased during pregnancy. The elevated estrogen levels were suggested to stimulate cholesterol uptake via increased LDLR expression as well as progesterone production via increased P450scc enzyme activity in cultured primary trophoblasts (Pepe and Albrecht, 1995; Grimes et al., 1996). In contrast, activities of the placental enzymes HMGR, ACAT, or CEH were found unaffected by estrogen (Babischkin et al., 1997a). This contrasts with estrogen-influenced HMGR regulation in other species and tissues (Trapani et al., 2010).

Recently, an impact of GDM on cholesterol synthesis and esterification in isolated and cultured human placental endothelial cells (HPECs) has been observed. The authors of the study suspected that higher intracellular ROS levels in GDM upregulated HMGR, increased *de novo* cholesterol biosynthesis and ACAT1 expression, but the underlying mechanisms remain to be identified (Sun et al., 2018).

Statins, inhibitors of HMGR, are increasingly prescribed to women of reproductive age (Forbes et al., 2015), but in experiments with first trimester placental explants or isolated first trimester trophoblasts, statins revealed detrimental effects on trophoblast growth (Forbes et al., 2008). Statins not only significantly decreased progesterone secretion (Kenis et al., 2005). They also inhibited proliferation (Forbes et al., 2015) and migration (Tartakover-Matalon et al., 2007) of trophoblasts. Isoprenylation, which also depends on HMGR activity (**Figure 3**), is required for cell proliferation, migration, metabolism, and protein glycosylation and thus, proper development of embryo and placenta. The existing data suggest that statins should be avoided during the first trimester of pregnancy (Ermini et al., 2017). On the other hand, there is evidence that statins, due to their anti-proliferative, anti-invasive, anti-inflammatory, and anti-angiogenic effects might be useful in treatment of various obstetric and gynecologic conditions including endometriosis, PCOS, ovarian cancer, preeclampsia, and antiphospholipid syndrome (Esteve-Valverde et al., 2018; Zeybek et al., 2018). Further studies are required to understand the mechanisms of action of statins during gestation. Moreover, clinical trials to investigate the efficacy, safety, and appropriate dosage of different statins during pregnancy are needed.

In summary, the regulation of placental cholesterol synthesis requires further characterization, but species-specific placental structure and function should be considered. Many of the involved enzymes are regulated at multiple levels (Shi et al., 1999). Besides total mRNA and protein levels, DNA- and protein modifications and protein activities should be analyzed to obtain conclusive information about the mechanism of regulation. Since placental tissue is a mixture of different cell types, analysis of purified cell populations should be preferred over total tissue analysis to decipher cell type-specific regulation of protein expression (Shi et al., 1999).

### Maternal Lipoprotein Particle Uptake by the STB

Fetuses produce a significant fraction of the required cholesterol via endogenous synthesis. In humans, genetic defects in *de novo* cholesterol synthesis result in severe congenital birth defects. SLOS is caused by a deficiency of DHCR7 (**Figure 3**), which catalyzes the conversion of 7-DHC to cholesterol (Jira, 2013; Kanungo et al., 2013). SLOS fetuses with null mutations in DHCR7 exhibit no endogenous cholesterol synthesis, but they have some cholesterol in tissues and blood at birth indicating placental cholesterol transfer (Tint et al., 1995).

Amount and period of materno-fetal cholesterol transport remain under debate. Extrapolation of data from non-human species is difficult as the quantity of cholesterol derived from the maternal circulation differs (Connor and Lin, 1967; Pitkin et al., 1972; Cavender et al., 1995; Woollett, 1996, 2005; Jurevics et al., 1997; Woollett and Heubi, 2000; Herrera, 2002) ranging from very low levels in rat (Belknap and Dietschy, 1988; Jurevics et al., 1997) to more than 40% in the rhesus monkey (Pitkin et al., 1972). Studies in humans revealed that materno-fetal cholesterol transfer occurred throughout pregnancy (Plotz et al., 1968; Hellig et al., 1970; Lin et al., 1977). However, mainly during early development maternal cholesterol serves as the primary source of fetal cholesterol (Napoli et al., 1997; Baardman et al., 2012).

**FIGURE 4:**
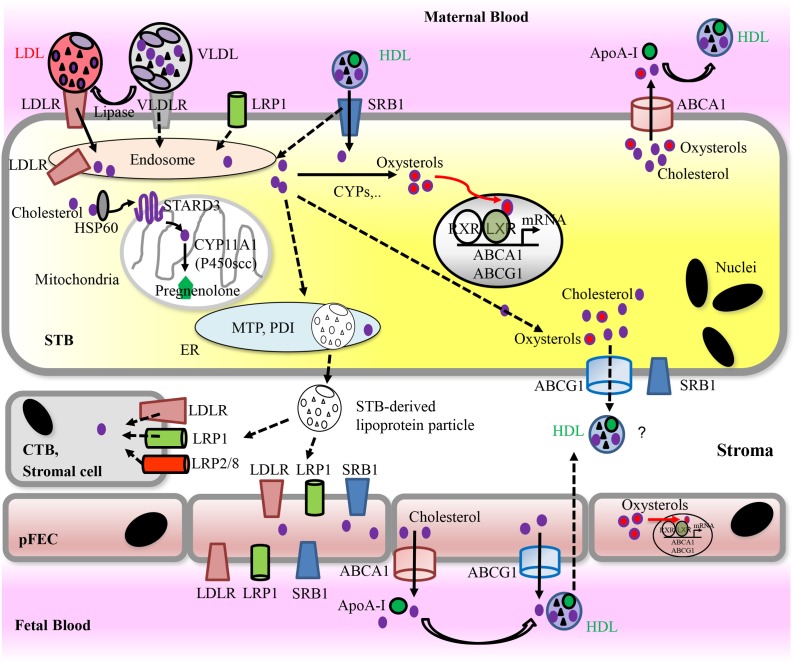
Proposed model for placental uptake of cholesterol from maternal lipoproteins, cholesterol metabolism, and materno-fetal transport of cholesterol. For detailed information, see text. Solid arrows indicate pathways that have been demonstrated *in vitro*. Dashed arrows indicate hypothetic routes. Apo, Apolipoprotein; ABC transporters, ATP-binding cassette transporters; CYPs, cytochrome P-450 enzymes; CTB, cytotrophoblast; pFECs, placental-fetal endothelial cells; HDL, high-density lipoprotein; HSP, heat shock protein; LDL, low-density lipoprotein; LDLR, low-density lipoprotein receptor; LRP, lipoprotein receptor-related proteins; LXR, liver X receptor; PDI, protein disulfide isomerase; MTP, microsomal triglyceride transfer protein; RXR, retinoid X receptor; SRB1, scavenger receptor class B type 1; STARD3, StAR-related lipid transfer domain protein 3; STB, syncytiotrophoblast; VLDL, very low-density lipoprotein; VLDLR, very low-density lipoprotein receptor.

Subsequently, we summarize the mechanisms of placental cholesterol uptake and transport (**Figure 4**). Cholesterol transport across the secondary yolk sac that may participate in nutrition of the human fetus during the first trimester (Burton et al., 2001) was reviewed elsewhere (Baardman et al., 2013).

In plasma, cholesterol is associated with different types of lipoprotein particles. Among them, LDLs carry 65–70% of circulating plasma cholesterol. Lipoprotein particles can interact with the plasma membrane of target cells via members of the LDLR family including LDLR, VLDLR, LRP1, LRP2 (megalin), or LRP8 (apoE receptor 2) (Go and Mani, 2012).

The presence of LDL-specific binding sites was shown throughout pregnancy in preparations of microvillous placental membranes, representing enriched apical STB plasma membranes (Alsat et al., 1982, 1984; Rebourcet et al., 1986; Naoum et al., 1987). Expression of LDLR mRNA in baboon STB increases with advancing pregnancy (Albrecht et al., 1995). Estrogen, but also depletion of cholesterol upregulate LDLR expression (Henson et al., 1991, 1997; Pepe and Albrecht, 1995; Grimes et al., 1996; Babischkin et al., 1997a,b). But conflicting data concerning the regulation of LDLR mRNA levels in total placental tissue in the course of human pregnancy exist (Furuhashi et al., 1989; Albrecht et al., 1995; Murata et al., 1996; Plosch et al., 2010). Additional expression of LDLR in stromal cells in the chorionic villi (Kamper et al., 2017) and cell-type specific receptor regulation may account for that discrepancy.

The transfer of lipids between HDL and target cells is incompletely understood. A number of proteins and receptors have been described to bind HDL. After receptor binding, HDL associated lipids are transferred to cells predominantly without catabolism of the particle. Transfer of cholesteryl esters to the accepting plasma membrane is known as “selective uptake” (Linton et al., 2017). An alternative pathway comprising endocytosis of whole HDL particles followed by resecretion exists. Neither the connection between HDL endocytosis and selective lipid uptake nor the physiological relevance of HDL uptake is fully clarified. SRB1 (in humans also termed CLA-1) is a multiple ligand receptor able to facilitate uptake of cholesteryl esters from HDL. SRB1 can also mediate whole HDL particle endocytosis (Rohrl and Stangl, 2013).

HDL binding-sites as well as SRB1 were found on isolated placental microvillous and basal plasma membranes of the STB (Alsat and Malassine, 1991; Lafond et al., 1999). *In situ*, SRB1 localizes to first and third trimester trophoblasts as well as term pFECs (Landers et al., 2018). Cultured first trimester trophoblasts expressed SRB1, exhibited selective cholesteryl ester-uptake from HDL_3_, but also displayed considerable HDL endocytosis and degradation. In term cultured trophoblasts, SRB1 expression was lower. Likewise, selective uptake as well as HDL degradation were decreased compared to first trimester cells, but were still elevated compared to other tissues (Wadsack et al., 2003). *In vitro*, SRB1 involvement in selective cholesteryl ester-uptake from HDL_3_ was demonstrated (Wadsack et al., 2003). In total placental tissue highest SRB1 expression levels were observed in term placenta (Plosch et al., 2010), which might be explained by the expression of SRB1 in pFECs (Stefulj et al., 2009) and the increase of the proportion of vessels from 37% in first trimester to 63% at term (Zhang et al., 2002). In pregnant women with high serum cholesterol levels, LDLR expression, but not SRB1 protein expression is down-regulated (Ethier-Chiasson et al., 2007). Cell-type specific, *SRB1* gene transcription can be regulated by hormones, including hCG and estrogen (Landschulz et al., 1996; Fukata et al., 2014). Whether and how SRB1 expression in human STB and pFECs is influenced by these hormones, remains to be determined.

VLDL binds to term placental microvillous membranes (Naoum et al., 1987) and the triglyceride-enriched VLDL is an important fatty acid supplier for the fetus. Hydrolysis of VLDL-associated triglycerides is mediated by lipases such as lipoprotein lipase, which are expressed at the apical membrane of the STB (Bonet et al., 1992; Lindegaard et al., 2005). While the placenta also expresses several receptors for apolipoprotein (Apo)E-enriched particles, including VLDLR (Gafvels et al., 1993; Wittmaack et al., 1995; Murata et al., 1996), LRP1 (Gafvels et al., 1992; Cao et al., 2014; Kamper et al., 2017), LRP2 (Burke et al., 2013; Kamper et al., 2017), and LRP8 (Kim et al., 1996; Ulrich et al., 2016), their involvement in cholesterol uptake by the STB has never been demonstrated. A mutation in human LRP2 gives rise to severe congenital anomalies in the offspring (Kantarci et al., 2007). This may indicate an important role of LRP2 in placental cholesterol transport, but LRP2 is a multiligand receptor and thus, may serve different roles in trophoblast biology (Coukos et al., 1994; Go and Mani, 2012).

### Impact of Diseases and Exogenous Compounds on Placental Lipoprotein Receptor Expression

GDM is a glucose intolerance, which is first recognized during pregnancy, but usually resolves after birth. In Europe, it has a prevalence of 5.4% (Eades et al., 2017). The most common adverse outcome is high fetal birth weight (macrosomia). Placental abnormalities include increased incidence of villous immaturity, increased measures of angiogenesis, and higher placental weight. GDM is also associated with a higher risk of maternal and fetal complications including development of type 2 diabetes in later life (Huynh et al., 2015; Gallo et al., 2016; Rani and Begum, 2016). In GDM, altered maternal and neonatal lipid profiles were observed. Moreover, expression of LDLR and SRB1 was induced (Dube et al., 2013).

IUGR is observed in 5–10% of pregnancies. Due to a compromised placental function, the fetus does not reach its intrauterine potential for growth and development. As the second leading cause of perinatal mortality IUGR is responsible for 30% of stillborn infants. Long-term health defects include impaired neurological and cognitive development and cardiovascular or endocrine diseases in adulthood (Nardozza et al., 2017). IUGR is associated with a decreased supply of nutrients and/or oxygen to the growing fetus. Placental LDLR expression was found increased, while SRB1 expression was decreased compared to healthy controls (Wadsack et al., 2007).

Preeclampsia is a multifactorial pregnancy-specific disease affecting 3–5% of pregnancies. It is defined by *de novo* hypertension manifested after 20 weeks of gestation in combination with either proteinuria (300 mg/day), maternal organ dysfunction or uteroplacental dysfunction (Tranquilli et al., 2014). IUGR and premature birth are clinically relevant complications. In addition, preeclampsia affects the long-term outcome of both mothers and their offspring (Bokslag et al., 2016). Serum levels of triglycerides, LDLs, small dense LDLs that are susceptible to oxidation, as well as oxLDLs are higher in women with preeclampsia compared to healthy pregnant women (Chigusa et al., 2013). Murata et al. (1996) found decreased expression of VLDLR and LDLR mRNA in third trimester placentas derived from preeclamptic women. Hentschke et al. (2013) reported no differences in LDLR, LRP1, SRB1 mRNA levels when comparing placentas of healthy and preeclamptic women. But they observed reduced LRP1 mRNA expression in placentas of preeclamptic mothers delivering small-for-gestational-age babies compared to healthy controls (Hentschke et al., 2013).

Maternal exposure to cadmium, which is present in tobacco smoke, is associated with low birth weight and possibly with an increased incidence of spontaneous abortion. Cadmium accumulates in the placenta and reduces progesterone secretion. Partly, this is related to a down-regulation of LDLR mRNA (Jolibois et al., 1999; Kawai et al., 2002).

In summary, the human placenta expresses several receptors for lipoprotein particles. For most receptors, their involvement in and individual contribution to placental cholesterol uptake remains unexplored. Moreover, regulation of receptor expression under physiologic and pathologic conditions of pregnancy has been barely investigated.

### Intracellular Cholesterol Transport in the STB

After internalization into acidic endosomal compartments, dissociation of receptor-ligand complexes occurs. The receptors return to the plasma membrane, while lipoprotein particles enter the lysosomal route for degradation. Cholesterol incorporates into the endosomal/lysosomal membranes by coordinated actions of NPC1 and NPC2. Cholesterol transport to mitochondria for steroidogenesis is achieved by lipid transfer proteins including StAR/STARD1 and other members of the START domain protein family (Elustondo et al., 2017). Cholesterol transport to other cellular targets, e.g., the plasma membrane occurs by vesicular and non-vesicular means, the latter involving cholesterol binding to various proteins (Miller and Auchus, 2011; Luo et al., 2017). Although expression of some intracellular cholesterol transporting proteins including NPCs, NPC1-Like1, ABC-transporters ABCA2, SCP-x, STARD3, and HSP60 has been demonstrated in human term placenta (Tuckey et al., 2004; Burke et al., 2009; Monreal-Flores et al., 2017), the mechanism of intracellular cholesterol transport in STB remains largely uncharacterized.

### Cholesterol Exit From the STB

To eliminate cholesterol, hepatocytes secrete lipoprotein particles (Sacks, 2015). Secretion requires ApoB and the ER-localized cofactor MTP. MTP transfers lipids to the forming lipoproteins. It is a dimer of a 97-kDa protein and PDI (Walsh and Hussain, 2017). Most other cell types release excess cholesterol to extracellular lipid acceptors. Mechanisms accounting for cholesterol efflux include passive diffusion as well as active pathways mediated by ABCA1, ABCG1, and SRB1. Several factors including cellular cholesterol status, lipid transporter activity, and the nature of extracellular acceptors, influence the efficiency of cholesterol efflux. ABCA1 and ABCG1 are specifically important for the elimination of cholesterol from cells and tissues and for the biogenesis of HDL. ABCA1 stimulates cholesterol efflux to lipid-free apolipoproteins, predominantly to apoA-I, but also ApoE. In contrast, ABCG1 promotes efflux of cholesterol and oxysterols to HDL. SRB1 can mediate cholesterol efflux from peripheral cells to HDL, but not to lipid-free apoA-I (Phillips, 2014; Favari et al., 2015).

Efflux of cholesterol from the basal side of the STB is unclear. It may occur via secretion of either lipoprotein particles or of cholesterol complexed with apolipoproteins. ApoB, MTP large subunit and PDI are expressed in the STB (Kamper et al., 2017) and unique lipoprotein particles containing ApoB and ApoA-I have been isolated from placental tissue (Park et al., 1988). Furthermore, secretion of ApoB-100-containing lipoprotein particles was shown from term placental biopsies (Madsen et al., 2004). More recently, apical and basal secretion of ApoB in polarized grown BeWo cells was demonstrated (Kamper et al., 2017). Cholesterol complexed with ApoB or lipoprotein particles and secreted from the basal membranes of the STB could provide cholesterol and other lipids not only to pFECs and consequently to the fetus, but also to CTBs as well as stromal cells of the chorionic villi given that these cells express the relevant apolipoprotein receptors. Indeed, *in situ* expression of lipoprotein receptors such as LDLR or LRP1 on placental cells including pFECs or LRP2 on the CTB was shown (Kamper et al., 2017). This would enable uptake of STB-derived lipoprotein-associated cholesterol. In support of the idea that intact lipoproteins reach stromal cells of the villi, a capability to hydrolyze VLDL-triglycerides has been demonstrated on isolated placental macrophages (Bonet et al., 1992). However, how placental macrophages or other stromal cells cover their cholesterol requirements is currently unknown.

Apical secretion of ApoE from the STB was suggested to facilitate uptake of maternal non-LDL lipoprotein particles (Rindler et al., 1991); likewise, apical secretion of ApoB, which was observed in BeWo cells (Kamper et al., 2017), could be of relevance for regulation or induction of cholesterol uptake by trophoblasts. Moreover, apical secretion of apolipoproteins may enable RCT from the STB via apically expressed ABCA1 or SRB1 (see below). However, these speculations require further investigation.

Alternatively, cholesterol can exit the STB via ABCA1 and ABCG1, which stimulate efflux of cholesterol and oxysterols (Aye et al., 2009). Both transporters exhibit high expression in the placenta (Albrecht et al., 2007; Stefulj et al., 2009; Aye et al., 2010; Bhattacharjee et al., 2010; Nikitina et al., 2011; Bloise et al., 2016). They localize to apical (ABCA1) and basal membrane (ABCG1) of the STB and to the luminal surface of the pFEC (Albrecht et al., 2007; Stefulj et al., 2009; Aye et al., 2010; Baumann et al., 2013), but contradictory localization data also exist (Bhattacharjee et al., 2010; Nikitina et al., 2011). In cultured trophoblasts, up- or down-regulation of ABCA1 and ABCG1 stimulated or reduced, respectively, cellular cholesterol efflux (Aye et al., 2010). The presence of SRB1 at the apical and basal membrane of the STB (Lafond et al., 1999) would allow for bidirectional transport of liposoluble molecules to and from lipoprotein particles (Cao et al., 1997). In polarized grown BeWo cells, basal efflux of cholesterol was highest to HDL, and was suggested to occur via either SRB1 (Schmid et al., 2003), or ABCG1 (Woollett, 2011). However, expression and subcellular localization of SRB1, ABCA1, and ABCG1 seem to differ between BeWo cells and primary trophoblasts (Woollett, 2011). In any way, an efflux of cholesterol from the basal membrane of the STB via ABCG1 or SRB1 requires appropriate cholesterol acceptor molecules within the stroma of the villi. This has not been described.

### Cholesterol Transport by pFECs

Uptake of cholesterol by pFECs remains unknown. Efflux of cholesterol via ABCA1 and ABCG1 to apolipoproteins or HDL particles, respectively, has been confirmed in isolated term HPECs, while SRB1 does not contribute to HDL-mediated cholesterol release (Stefulj et al., 2009; Sun et al., 2018). Appropriate cholesterol acceptor molecules (HDL and ApoE) are available in the fetal circulation (Bansal et al., 2005).

### Regulation of ABC-Transporters via LXR in Health and Diseases

LXRs are “sterol sensors” responsible for protecting cells from cholesterol overload. LXR-activation by oxysterols (see below), but not cholesterol, induces transcriptional activity of the LXR/RXR heterodimer. This results in reduced intracellular cholesterol synthesis as well as cholesterol uptake, but induced expression of molecules implicated in RCT including ABCA1, ABCG1, and ApoE (Zhao and Dahlman-Wright, 2010; Soffientini and Graham, 2016; see **Figure 3**). Although oxysterols are generally perceived as endogenous agonists of LXR, some can act as antagonists (Mutemberezi et al., 2016). Due to their role in lipid metabolism, LXRs are considered as relevant drug targets. The synthetic ligands T0901317 and GW3965 are important tools in biomedical research. Unfortunately, they have poor LXR subtype selectivity and T0901317 is not only an LXR ligand, but also displays agonistic effects on farnesoid X receptor and pregnane X receptor (Zhao and Dahlman-Wright, 2010).

LXR-α and LXR-β as well as RXR can be detected throughout gestation, with increased expression of LXRs in preterm and term placentas (Marceau et al., 2005; Plosch et al., 2010). Preeclampsia or GDM were found to influence expression of LXRs in some (Weedon-Fekjaer et al., 2010b), but not all studies (Plosch et al., 2010).

LXR controls expression of ABCA1 and ABCG1 in cultured trophoblasts (Aye et al., 2010) and HPECs (Stefulj et al., 2009; Sun et al., 2018). Oxysterols or T0901317 increase cholesterol efflux, while LXR-inhibitors or siRNA-mediated LXR knock down decrease cholesterol efflux. As key regulators of the lipid metabolism, LXRs also play a regulatory role in fatty acid metabolism in trophoblast cells (Weedon-Fekjaer et al., 2010a).

Recently, upregulation of ABCA1 and ABCG1 in HPECs derived from GDM placentas compared to control placentas was shown. Upregulation was the consequence of GDM-induced increased ROS-formation, increased ROS-derived oxysterol levels and subsequent LXR activation. The resulting enhanced cholesterol efflux protected the cells from cholesterol overload due to GDM-induced increased cholesterol biosynthesis. Thus, the LXR-mediated upregulation of ABC-transporters in GDM appeared to restore cholesterol homeostasis (Sun et al., 2018). Alterations of expression levels of ABCA1 and ABCG1 in total placental tissue in case of pregnancy diseases such as GDM, preeclampsia, hypoxia, or antiphospholipid-syndrome were reported, but the results are partly inconsistent (Albrecht et al., 2007; Plosch et al., 2007, 2010; Korner et al., 2012; Baumann et al., 2013; Chigusa et al., 2013; Dube et al., 2013; Liu et al., 2014; Huang et al., 2018).

ABCA1 and ABCG1 also efflux oxysterols (Stefulj et al., 2009; Aye et al., 2010; Sun et al., 2018). In cultured trophoblasts, both transporters were shown to prevent accumulation of the oxysterols 25-hydroxycholesterol (25-OHC) and 7-ketocholesterol (**Figures 2C,E**), which exhibit cytotoxic potential at higher concentrations (Aye et al., 2010). ABCA1 expression increases during trophoblast syncytialization *in vitro* (Keelan et al., 2011). ABCA1 localization at the apical STB membrane *in situ* (**Figure 4**) may ensure efflux and thus placental elimination of oxysterols into maternal blood.

### Oxysterols and Pregnancy

Many different oxysterols have been identified; they are formed by either free radical oxidation or by enzyme-mediated mechanisms (Mutemberezi et al., 2016). Oxysterols exhibit multifaceted functions. Some oxysterols are important regulators of cholesterol homeostasis (**Figures 3**, **4**). 27-OHC (**Figure 2D**), produced from cholesterol by the enzyme CYP27A1, is the most prominent oxysterol in the bloodstream of human adults (Bjorkhem, 2002). *In vitro*, 27-OHC inhibits cholesterol synthesis by negative feedback regulation of HMGR. 27-OHC is an agonist of LXR stimulating cholesterol efflux from cells. Increased CYP27A1 and/or 27-OHC levels might reflect the attempt to remove excess cholesterol from cells and to limit lipid peroxidation. Some studies show that diseases of pregnancy such as preeclampsia are associated with altered expression of placental CYP27A1 protein and altered levels of 27-OHC in placenta, maternal, and/or fetal serum. But these studies are partly contradictory and the mechanism behind the alteration and the impact of these changes on placental cholesterol metabolism remains to be determined (Moon et al., 2014; Mistry et al., 2017; Winkler et al., 2017).

Oxysterols are major components of oxLDL. They contribute to the pathophysiology of atherosclerosis, and are found at increased levels in atherosclerotic lesions (Vejux et al., 2008; Levitan et al., 2010). OxLDL serum levels rise even during normal gestation (Belo et al., 2004; Makedou et al., 2011). Elevated levels of oxLDL and oxysterols have been identified in maternal serum in preeclampsia, pregnancy-induced hypertension, GDM, and diabetes mellitus type I as well as in the cord blood of neonates with IUGR or GDM (Bodzek et al., 2002a,b; Uzun et al., 2005; Qiu et al., 2006; Kim et al., 2007; Leduc et al., 2011; Moon et al., 2014; Sun et al., 2018). Overall, the cytotoxic and pro-inflammatory activities of oxysterols contribute to the development of many diseases associated with oxidative stress (Vejux et al., 2008; Zarrouk et al., 2014).

Oxysterols can influence various aspects of trophoblast function. The oxysterols 25-OHC and 7-keto-cholesterol (**Figures 2C,E**), which are present in oxLDLs, reduce trophoblast invasion via activation of LXR (Pavan et al., 2004; Fournier et al., 2008). Moreover, 25-OHC inhibits syncytialization of CTB (Aye et al., 2011). Oxysterols increase the release of soluble endoglin, a molecule present at elevated concentrations in the maternal circulation in women with preeclampsia, via increased expression of matrix metalloproteinase 14 in the placenta (Valbuena-Diez et al., 2012; Brownfoot et al., 2014). Oxysterols promote the production of pro-inflammatory cytokines in placental trophoblasts, via activation of TLR 4 thus providing a mechanistic link between oxidative stress in pregnancy and placental inflammation. This pro-inflammatory action of oxysterols predominated over simultaneously observed anti-inflammatory effects mediated by oxysterols via LXR activation (Aye et al., 2012). It has been speculated that LXR activation indirectly supports the repression of TLR target genes activation. LXR activation results in increased placental expression of ABCA1 and ABCG1 (Stefulj et al., 2009; Aye et al., 2010; Sun et al., 2018). This may change cellular oxysterol levels as well as alter the cholesterol content of the membrane microdomains required for TLR signaling by promoting cholesterol efflux.

The dual (pro-inflammatory/anti-inflammatory) role of oxysterols observed in trophoblast cells (Aye et al., 2012) already indicates the complexity associated with these molecules. In addition, a study by Larkin et al. (2014) revealed that the effects of oxysterols on trophoblasts are concentration dependent. 25-OHC applied at higher (100 μM) concentrations was found to be cytotoxic, to inhibit differentiation as well as progesterone secretion of trophoblast cells. In contrast, low (10 μM) concentrations stimulated differentiation, progesterone secretion and ABCA1 expression, and reduced SRBP2, LDLR and HMGR expression (Larkin et al., 2014). To add to the complexity, not all of these cellular effects are mediated via LXR (Larkin et al., 2014). Oxysterols are known to interact with many proteins. RORα and RORγ are other nuclear receptors binding oxysterols. RORs are transcription activators and RORγ was shown to regulate hepatic lipid metabolism. Loss of RORγ reduces the expression of a number of lipid metabolic genes, which in turn reduces the levels of triglycerides, cholesterol, and bile acids in liver and blood. 25-OHC is an agonist of RORγ, while other oxysterols may function as inverse agonists (Mutemberezi et al., 2016; Jetten et al., 2018). Oxysterols including 25-OHC are able to bind INSIGs, which induces interaction of INSIGs and SCAP and consequently inhibits SREBP activation. 25-OHC can regulate cholesterol synthesis by interaction with NPC1 and 2. Finally, 25-OHC can bind to OSBP and ORPs and modify their function (**Figure 3**). ORP8 can modulate ABCA1 expression and thereby influence cholesterol efflux (Mutemberezi et al., 2016).

To conclude, oxysterols can compromise placental formation, regeneration, and function, but also are important regulators of placental cholesterol metabolism and probably other physiological processes. Their effect may depend on the identity and concentration of the local oxysterol compound and the available intracellular binding partners. ABCA1 and ABCG1 were shown to prevent the toxic effects of oxysterols on placental and fetal development and function, and reduce the risks associated with diseases of pregnancy such as GDM. But altered regulation of these receptors or oxysterol binding partners in the case of pregnancy-associated diseases in combination with increased levels of oxysterols might (further) compromise placental function.

Our knowledge of function and regulation of oxysterols in the context of pregnancy and regulation of cholesterol metabolism remains limited. We need to explore the types of oxysterols induced during healthy and diseased pregnancy, and learn whether they are generated mainly by enzymes or by autoxidation. It remains to be determined whether they act primarily early (around implantation) or late in pregnancy. Published data are often contradictory. This might be related to problems associated with reliable measurements of oxysterols that are quite susceptible to autoxidation and the fact that oxysterol production is influenced by several factors including cell type, mode of experimentation or circadian variations (Schroepfer, 2000; Mutemberezi et al., 2016).

## Steroid Hormones

Steroid hormones comprise sex steroids and corticosteroids. In adults, the sex steroids – progestagens, estrogens, and androgens – are produced in ovaries and testes, while the corticosteroids – glucocorticoids and mineralocorticoids – are released from the adrenal cortex (Miller and Auchus, 2011; Miller, 2017). During pregnancy, the placental STB becomes a major source of progesterone (at term around 250 mg/day) and estrogens (at term around 100–120 mg/day). Fetal organs (adrenal cortex and liver), in contrast, synthesize corticosteroids and the androgens DHEA, DHEA-S, 16α-hydroxy-DHEA, and 16α-hydroxy-DHEA-S (Evain-Brion and Malassine, 2003; Costa, 2016; Pasqualini and Chetrite, 2016). These hormones are transfered between placental and fetal compartment and are subjected to transformation. Partly, fetus and placenta have complementary enzyme activities and thus are interdependent. Moreover, the hormones also reach the maternal compartment. For more information about steroid hormone exchange between fetus, placenta and mother the reader is referred to Pasqualini and Chetrite (2016). Proper function and interaction of the steroid producing tissues and the involved enzymes and transporters is crucial since the intrauterine exposure of the offspring to abnormal levels of glucocorticoids (Miranda and Sousa, 2018) or sex steroids (Cardoso et al., 2015; Pluchino et al., 2016) can negatively impact fetal development. Furthermore, abnormal concentrations of steroid hormones during pregnancy can increase the maternal risk for malignant diseases (Schock et al., 2014). The following chapters summarize the mechanisms of uptake, synthesis, and transformation of steroid hormones at the human placental barrier and highlight circumstances that cause alterations.

Steroid hormones are derived from cholesterol and thus have closely related structures based on the common steroid skeleton (**Figure 2A**). The major classes of enzymes required for the production of steroid hormones are the CYP heme-containing proteins (P450 and CYPs) and the HSDs. CYPs important for steroid hormone synthesis are CYP11A1, which is a mitochondrial protein, as well as CYP17A1, CYP19A1, and CYP21A2, all located in the ER. CYPs catalyze the hydroxylation and cleavage of steroid substrates, while reduction and oxidation of steroid hormones are effected by isoforms of 3βHSDs and 17βHSDs. Cell-type specific, HSDs localize to the membranes of either mitochondria or ER (Payne and Hales, 2004; Miller and Auchus, 2011). The major placental transporter families are SLCs and the ABC-transporters. SLCOs usually mediate influx of their substrates. They participate in uptake of hydrophilic or charged molecules such as sulfated steroids. In contrast, ABC-transporters mediate the efflux of substrates (Joshi et al., 2016; Walker et al., 2017).

### Sex Steroids and the Placenta

#### Progesterone

Progesterone (P4) is the major and most relevant progestagen. In humans, the ovarian corpus luteum secretes progesterone until week 8 of gestation, thereafter, the placenta completely takes over the production (Malassine et al., 2003).

**FIGURE 5:**
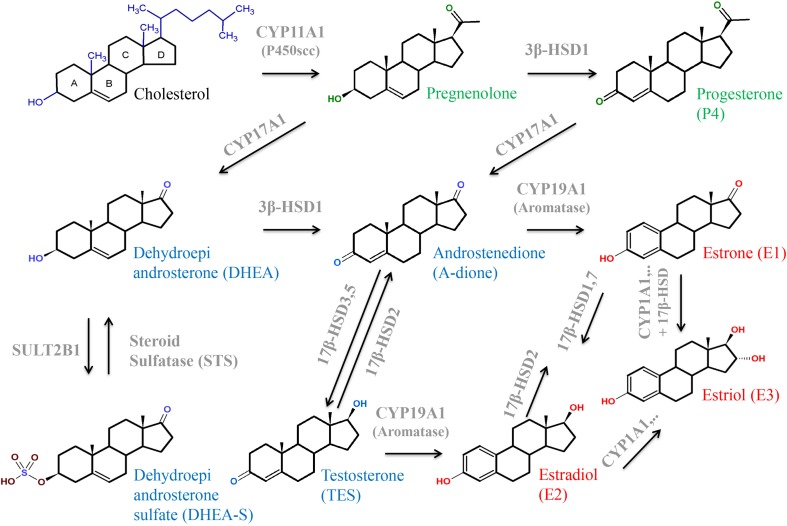
Structures and interconversion of the sex-steroids progestagens (green), estrogens (red), and androgens (blue) starting from cholesterol. For detailed information, see text. Arrows indicate metabolizing processes by respective enzymes (gray). CYPs, cytochrome P-450 enzymes; HSD, hydroxysteroid dehydrogenase; SULT, sulfotransferase.

**FIGURE 6:**
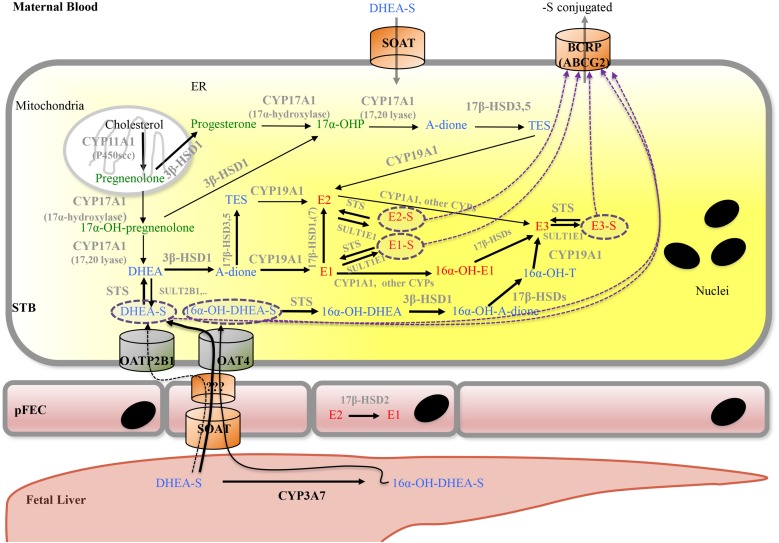
Proposed model for placental progesterone and estrogen synthesis. For detailed description, see text. Progestagens are shown in green, estrogens in red, and androgens in blue color. Solid arrows indicate enzymatic steps that have been demonstrated in human placenta; respective enzymes are shown in gray. Dashed arrows indicate hypothetic routes of transport of sulfate-conjugated compounds. Dashed purple circles highlight sulfate-conjugated compounds that require transporters for uptake into (SOAT, OAT4, and OATP2B1) and export (ABCG2) from cells. A-dione, androstenedione; ABC transporters, ATP-binding cassette transporters; BCRP, breast cancer resistance protein; DHEA, dehydroepiandrosterone; DHEA-S, dehydroepiandrosterone sulfate; CYPs, cytochrome P-450 enzymes; E1, estrone; E1-S, estrone sulfate; E2, Estradiol; E2-S, estradiol sulfate; E3, estriol; E3-S, estriol sulfate; ER, endoplasmatic reticulum; pFEC, placental-fetal endothelial cell; -OH-, -Hydroxy-; 17α-OHP, 17α-hydroxyprogesterone; HSD, hydroxysteroid dehydrogenase; OAT4, organic anion transporter 4; OATP2B1, organic anion transporting polypeptide 2B1; SOAT, sodium-dependent organic anion transporter; STS, steroid sulfatase; SULT, sulfotransferase; STB, syncytiotrophoblast; TES, testosterone.

Progesterone exerts many functions. Briefly, it is an intermediate in the production of other steroid hormones (**Figures 5**, **6**) and a neurosteroid involved in brain function. Progesterone is crucial for a successful pregnancy as it supports blastocyst implantation, maintains pregnancy, and prepares the mammary glands for lactation (Miller and Auchus, 2011; Halasz and Szekeres-Bartho, 2013; Costa, 2016; Di Renzo et al., 2016). Progesterone and synthetic progestins are successfully used for the prevention of preterm birth and for treatment of various gynecological disorders (Di Renzo et al., 2012, 2016).

The action of progesterone on target tissues is mediated by two progesterone receptor isoforms (A and B) that function as ligand-activated transcription factors. Under physiological conditions, the withdrawal of progesterone receptor-mediated signaling triggers menstruation and parturition (Wetendorf and DeMayo, 2012, 2014; Patel et al., 2015). Likewise, pathologic deregulation of the progesterone-receptor signaling pathway is associated with preterm delivery (Patel et al., 2015; Tiwari et al., 2016). For example, poor pregnancy outcome due to hepatitis E virus infection is related to low expression of functional progesterone receptors and high expression of receptor haplotypes exhibiting reduced response to progesterone (Romano et al., 2007; Bose et al., 2011).

#### Physiology and Pathophysiology of Progesterone Synthesis

Synthesis of progesterone starts within mitochondria (**Figures 4**, **6**). Cholesterol has to be loaded into the outer mitochondrial membrane in a process that is not entirely understood (Miller and Auchus, 2011; Miller, 2017). In most steroidogenic tissues, the transport of cholesterol from the outer to the inner mitochondrial membrane is mediated by StAR, which can be acutely stimulated by tissue-specific trophic hormones (Miller, 2013); however, StAR is not expressed in human placenta (Sugawara et al., 1995). Instead, StAR-like protein, STARD3 (or MLN64) (Bose et al., 2000; Tuckey et al., 2004) as well as HSP60 are involved in mitochondrial cholesterol import in the placenta (Tuckey, 2005; Olvera-Sanchez et al., 2011; Esparza-Perusquia et al., 2015; Monreal-Flores et al., 2017). In JEG-3 cells, HSP60 additionally participates in delivery of cholesterol to the metabolizing enzyme CYP11A1 (Monreal-Flores et al., 2017). STARD3 action requires a complex multicomponent molecular machine on the outer mitochondrial membrane, which includes, among other factors, the translocator protein TSPO. In obese women (BMI 38.8 ± 6.4 kg/m^2^), who exhibit altered insulin sensitivity and leptin level, the serum levels of progesterone as well as estradiol were significantly reduced. While expression of key enzymes of placental progesterone and estrogen synthesis (CYP11A1, 3β-HSD1, and 17β-HSD1) was not altered, the expression levels of TSPO as well as the cholesterol content in placental mitochondria were decreased. *In vitro*, long chain fatty acids and LPS could reduce TSPO expression (Lassance et al., 2015).

After mitochondrial import, cholesterol is hydroxylated at two positions (C-20 and C-22) and the cholesterol side chain is cleaved off by P450 side-chain cleaving enzyme (P450scc or CYP11A1; **Figures 5**, **6**; Hochberg et al., 1974; Slominski et al., 2015b). CYP11A1 is a rate-limiting enzyme of steroidogenesis and converts cholesterol to pregnenolone, the precursor of all other steroid hormones (Hanukoglu, 1992; Miller and Auchus, 2011; Miller, 2013). Moreover, CYP11A1 is also a metabolizing enzyme for other sterols. It mediates conversion of 7-DHC to 7-dehydropregnenolone and hydroxylates the side chain of vitamin D (Slominski et al., 2005, 2006, 2009, 2012, 2015a,b).

CYP11A1 localizes exclusively to the STB (Li et al., 2005; He et al., 2013). The placental CYP11A1 protein expression level remain constant from first to term trimester (Henderson et al., 2008; He et al., 2013). The major regulatory factor for *CYP11A1* gene expression in other steroidogenic tissues, SF-1, is absent in the human placenta. Instead, human placental *CYP11A1* gene expression is tightly regulated by one activator (LBP-1b) and two repressor proteins (LBP-9 and LBP-32). In analogy to CYP11A1, these proteins are expressed already very early in pregnancy and localize to the STB (Henderson et al., 2008).

CYP11A1 is upregulated at both mRNA and protein level in placentas of women with severe preeclampsia. Overexpression of CYP11A1 reduces trophoblastic cell proliferation and induces apoptosis in the HTR8/SVneo extravillous trophoblast cell line through activation of caspase-3 expression (Enquobahrie et al., 2008; He et al., 2013). In line with increased expression of CYP11A1 during preeclampsia, the level of pregnenolone (and progesterone) is increased in preeclamptic women (Moon et al., 2014). CYP11A1 is also involved in lipid peroxidation. Based on results obtained in isolated placental mitochondria and JEG-3 cells, it has been speculated that not only excess progesterone synthesis but also production of lipid peroxides by increased expression of placental CYP11A1 may contribute to the pathogenesis of preeclampsia (Zabul et al., 2015).

In contrast, life style factors like smoking or cocaine may inhibit progesterone synthesis. Cadmium, which is found in cigarette smoke, reduces CYP11A1 mRNA expression as well as progesterone synthesis in human cultured trophoblasts (Kawai et al., 2002). A study in pregnant rats treated with cocaine demonstrated significantly reduced maternal serum levels of pregnenolone and progesterone as well as reduced mRNA and protein expression of CYP11A1 and STARD3 (Wu et al., 2012).

Pregnenolone is converted to progesterone by 3β-HSD1 in the ER (**Figures 5**, **6**; Miller and Auchus, 2011; Liu et al., 2016). 3β-HSD1 is exclusively expressed in the placental STB (Ian Mason, 1993; Li et al., 2005), while 3β-HSD2 is predominantly expressed in the adrenal gland and gonads (Labrie et al., 1992; Ian Mason, 1993). Besides pregnenolone, human 3β-HSD1 can also use 17α-hydroxypregnenolone and DHEA as substrates (Hanukoglu, 1992). Various endogenous hormones including estradiol, insulin, insulin-like growth factor, calcitriol, leptin, and CRH can modulate progesterone synthesis (Costa, 2016). Unfortunately, also environmental toxins influence activity of 3β-HSD1 and thus progesterone synthesis. Evidence for this was obtained in the choriocarcinoma cell line JEG-3. Fine PM (<2.5 μm, PM2.5) is a leading air pollutant and exposure to PM2.5 during the prenatal period increases the risk of adverse pregnancy outcome. Exposure of cells to PM2.5 causes reduced progesterone secretion as well as reduced expression of 3β-HSD1 and CYP11A1 mRNA and protein (Wang et al., 2017). The fungicide tributyltin lowers progesterone production and acts as a moderate inhibitor of 3β-HSD1 (Cao et al., 2017). Likewise, the insecticides methoxychlor and its metabolite hydroxychloroquine inhibit progesterone as well as estradiol production; these substances are potent 3β-HSD1 inhibitors (Liu et al., 2016). In contrast, ochratoxin A, a common food-borne mycotoxin, induces the expression of 3β-HSD1, leading to a significant increase of progesterone production (Woo et al., 2013).

#### Estrogens

The group of estrogen steroids comprises estrone (E1), estradiol, (E2), estriol (E3), and estetrol (E4). Among these, estradiol is the most abundant estrogen. Estrogen’s multiple functions have been summarized on several occasions and include stimulation of utero-placental blood flow, endometrial growth and differentiation, contraction of the myometrium and proliferation of the mammary epithelium (Mesiano, 2001; Costa, 2016). As detailed in the chapter on cholesterol, estrogens also regulate expression of genes required for placental cholesterol supply. They exert their effect via different types of receptors, the nuclear estrogen receptors α and β as well as membrane-associated receptors. The synthesis of estrogens is controlled by diverse endogenous hormones such as estradiol, cortisol, calcitriol, CRH, hCG, insulin, and leptin (Costa, 2016).

#### Physiology and Pathophysiology of Estrogen Synthesis

Synthesis of estrogens involves several enzymes and transporters (**Figures 5**, **6**). CYP17A1 (or P450c17) is a bifunctional enzyme with 17α-hydroxylase and 17,20-lyase activities that converts pregnenolone to 17α-hydroxypregnenolone and subsequently to DHEA. Alternatively, progesterone can be converted to 17α-hydroxyprogesterone (17α-OHP) and then to A-dione (Escobar and Carr, 2011; Thomas and Potter, 2013). Early studies in human placentas and choriocarcinoma cell lines could not demonstrate placental CYP17A1 activity or mRNA expression (Siiteri and MacDonald, 1966; Bahn et al., 1981; Voutilainen and Miller, 1986). The concept emerged that the placenta was unable to convert pregnenolone and progesterone to the androgen products. Thus, compulsive import of sulfated fetal C19-androgens, 16α-hydroxylated DHEA-S and DHEA-S, which are further processed into estrogens by placental STS, 3βHSD1, CYP19A1 (aromatase), and 17βHSD isoenzymes, was assumed. However, more recent studies (Pezzi et al., 2003; Escobar et al., 2011; Noyola-Martinez et al., 2017) demonstrated placental CYP17A1 mRNA expression, although at a much lower level as compared to 3β-HSD1, CYP19A1, CYP11A1, and 17β-HSD3. CYP17A1 protein was detected in placental STB and JEG-3 cells (Escobar et al., 2011). 17α-OHP synthesis by CYP17A1 in trophoblasts is regulated by the cAMP/PKA pathway (Escobar and Carr, 2011). The authors estimated that 20–30% of the estrogen produced during pregnancy could result from pregnenolone conversion by placental CYP17A1.

While endogenous production of DHEA in the STB occurs, a large fraction of the sulfo-conjugated C19-estrogen precursors 16α-OH-DHEA-S and DHEA-S is derived from the fetus (**Figure 6**); maternal DHEA-S is also used, but to a minor extent (Kallen, 2004; Pasqualini and Chetrite, 2016). DHEA is produced in the fetal adrenal gland, which is also rich in SULT activity, thus generating DHEA-S. 16α-OH-DHEA-S, that is the most abundant estrogen precursor, is synthesized in the fetal liver by 16α-hydroxylation of DHEA-S via CYP3A7 (Leeder et al., 2005). Fetal hepatic CYP3A7 expression is detected around day 50 to 60 of gestation (Yang et al., 1994); expression of CYP3A7 in human placenta remains unclear (Hakkola et al., 1996; Maezawa et al., 2010). Unconjugated estrogens, synthesized by the placenta, are released into both the maternal and fetal blood by diffusion. In part, they get sulfated in the maternal and fetal compartment and re-enter the placenta by carrier-mediated transport (see below). Moreover, the human placenta exhibits STS activity as well as SULT activities (e.g., SULT1E1 and SULT2B1) (Stanley et al., 2001; Miki et al., 2002; He et al., 2004) and thus can convert unconjugated estrogens (or other substrates) into sulfated forms and *vice versa*, respectively. For more information on the function of sulfated steroid hormones in reproduction, the reader is referred to Geyer et al. (2017).

Uptake of conjugated fetal and maternal steroids requires placental expression of appropriate transport systems (Geyer et al., 2017). The human SLC family 22 member 11 (SLC22A11) also known as OAT4, is predominantly expressed in placenta and kidney (Cha et al., 2000). In placenta, OAT4 was detected at the basal plasma membrane of the STB as well as on the CTB in first- and third-trimester placentas (Ugele et al., 2003; Noguchi et al., 2015; Tomi et al., 2015). Studies using isolated basal membrane fractions of placental STB, primary trophoblasts, JEG-3 cells as well as OAT4-transfected cell lines demonstrated uptake of sulfated steroids, including DHEA-S, 16α-OH-DHEA-S and sulfated estrogens via OAT4 (Cha et al., 2000; Ugele et al., 2008; Schweigmann et al., 2014; Tomi et al., 2015). PKA regulates OAT4-mediated transport of sulfated steroids (Tomi et al., 2014). The SLC OAT family member 2B1 (SLCO2B1, OATP2B1, and OATP-B) is also expressed at the basal surface of the STB and on the CTB (St-Pierre et al., 2002). However, 16α-OH-DHEA-S is not a substrate of SLCO2B1. SLCO2B1 can transport sulfated estrogens, while conflicting data exist about whether DHEA-S is a substrate of this transporter (Grube et al., 2007; Ugele et al., 2008; Schweigmann et al., 2014). SLCO2B1 activity is regulated by unconjugated steroid hormones (Grube et al., 2006). The BCRP (ABCG2) is found on the apical surface of placental STB (Nakamura et al., 1997). Placental ABCG2 appears regulated by several endogenous factors (including hormones) as well as exogenous factors (Hahnova-Cygalova et al., 2011). In ABCG2/OATP2B1-overexpressing epithelial MDCKII cells, Grube et al. (2007) obtained evidence that OATP2B1 and ABCG2 together mediate basolateral-to-apical directed transport of the steroid sulfates E3-S and DHEA-S (Grube et al., 2007). Thus the current concept for uptake of fetal sulfated steroid is that OAT4 can transport sulfo-conjugated estrogens as well as sulfated C19-steroid precursors for placental *de novo* synthesis of estrogens. In contrast, OATP2B1 (in combination with ABCG2) may rather contribute to the clearance of estrogen sulfates (as well as DHEA-S?) from the fetal circulation (Ugele et al., 2003; Grube et al., 2007). SOAT was identified as transporter for both 16α-OH-DHEA-S and DHEA-S (Schweigmann et al., 2014). SOAT was detected in the apical membrane of STB as well as on pFECs in the third trimester of pregnancy (Schweigmann et al., 2014). This transporter may thus mediate uptake of fetal 16α-OH-DHEA-S and DHEA-S by pFECs and maternal DHEA-S by the STB. A mechanism for exit of sulfated steroids at the abluminal side of pFECs is unknown (**Figure 6**).

STS, also known as aryl sulfatase C is responsible for hydrolysis of steroid sulfates such as DHEA-S, 16α-OH-DHEA-S, E1-S, E2-S, and E3-S, leading to the production of their unconjugated active forms (Thomas and Potter, 2013). STS is expressed in the STB (French and Warren, 1965; Miki et al., 2002; Suzuki et al., 2003) and STS mRNA and protein is significantly elevated in placentas from early onset preeclamptic women (Gratton et al., 2016). In preeclampsia, anti-angiogenic factors such as soluble fms-like tyrosine kinase (sFlt)1 disrupt the maternal endothelium by binding circulating angiogenic factors, which causes the symptomatic second stage of preeclampsia including dysregulated placental perfusion and ischemia (Maynard et al., 2003). Silencing of STS in primary placental trophoblasts resulted in a significant decrease in sFlt1 secretion and a significant reduction in sFlt1 transcription (Gratton et al., 2016). Thus, it was speculated that high STS expression could contribute to preeclampsia via altered sFlt1 regulation (Gratton et al., 2016).

3β-HSD1 catalyzes the conversion of DHEA into A-dione, which is further transformed to estrone by the P450 aromatase (CYP19A1). CYP19A1 could also convert testosterone into estradiol (Hanukoglu, 1992; Thomas and Potter, 2013), but affinity of CYP19A1 for A-dione is much higher (Yoshida and Osawa, 1991). Placental CYP19A1 expression and function are diminished in pregnancies complicated by preeclampsia compared to controls (Perez-Sepulveda et al., 2015). PCOS is a common endocrinal metabolic disorder, affecting approximately 5–10% of women at reproductive age. It is characterized by ovulatory/menstrual irregularity, polycystic ovaries, and hyperandrogenism, including progesterone resistance (Goodarzi et al., 2011; Piltonen et al., 2015). In placental tissue from women with PCOS, reduced activities of CYP19A1 and increased activities of 3β-HSD1 were observed when compared to control women. Moreover, women with PCOS showed higher A-dione and testosterone concentrations compared to normal pregnant women (Maliqueo et al., 2013).

17β-HSDs are also known as 17-ketosteroid reductases. They catalyze the reversible conversion of 17-keto and 17β-hydroxy groups in androgens and estrogens, including A-dione, DHEA, and estradiol. The direction of the reaction depends on the substrate (Hanukoglu, 1992; Thomas and Potter, 2013). 17β-HSD1 predominantly catalyzes the NADPH-promoted stereospecific reduction of estrone to the more active estradiol (Thomas and Potter, 2013; Herman et al., 2016). 17β-HSD2 shows oxidative activity and is capable of catalyzing the conversion of estradiol, testosterone, and dihydrotestosterone to their less-active 17-keto forms, estrone, A-dione, and 5α-androstanedione, respectively (Rantakari et al., 2008). These two enzymes were found to have different locations in the placenta. While 17β-HSD1 was detected in the STB already at week 4 of gestation, 17β-HSD2 was expressed in the pFEC and detected only after week 12 of gestation. 17β-HSD2 was suggested to prevent the excessive passage of active estrogens into the fetal circulation by catalyzing the inactivation of estradiol to estrone within the pFECs (Takeyama et al., 1998; Bonenfant et al., 2000; Drolet et al., 2007). Placental expression of 17β-HSD3, which is involved in testosterone formation, is increased in preeclamptic women (Shao et al., 2017). 17β-HSD5 protein (Phillips et al., 2014) and 17β-HSD7 mRNA (Krazeisen et al., 1999) expression were shown in human placenta.

Estriol is mainly synthesized from 16α-OH-DHEA-S, but, alternatively, can be converted from estrone or estradiol. This requires expression of enzymes with 16α-hydroxylase activity such as CYP1A1 (Duttaroy and Basak, 2015; **Figure 6**).

Overall, in preeclampsia, and specifically early onset preeclampsia, several of the placental enzymes involved in progesterone and estrogen formation and transformation show altered expression. Moreover, the placental expression levels of steroid receptors (estrogen receptor α and β, progesterone receptor) can change in preeclampsia (Park et al., 2018). Whether the alterations are a consequence of preeclampsia or precede the disease is not known. Maternal serum progesterone and estrogen levels are found reduced, while androgen levels are increased in preeclampsia (Ghorashi and Sheikhvatan, 2008; Hertig et al., 2010; Bussen and Bussen, 2011; Sharifzadeh et al., 2012; Acikgoz et al., 2013; Moon et al., 2014; Perez-Sepulveda et al., 2015; Shao et al., 2017; Shin et al., 2018; Wan et al., 2018). But data remain partly conflicting, which might be due to diverging characteristics of the patients selected.

Most of the reports are mainly descriptive without any analysis of mechanisms causing the underlying changes. One exception is a recent study describing a significant up-regulation of microRNA (miR-22) in placentas derived from preeclamptic women (Shao et al., 2017). MicroRNAs (miRNAs) are small non-coding RNAs of about 22 nucleotides in length that play a critical role in post-transcriptional gene regulation (Bartel, 2009). The authors demonstrated significantly increased testosterone and reduced estradiol levels in plasma samples as well as increased placental expression levels of 17β-HSD3 and reduced placental aromatase expression in women with early onset preeclampsia. Furthermore, increased levels of the miR-22 were detected mainly in placental villous and extravillous trophoblasts. JEG-3 cells were then used to explore the mechanism behind the changes. Increasing testosterone concentrations repressed the expression of aromatase and estrogen receptor α and the production of E2. Testosterone caused increased miR-22 expression, which directly inhibited estrogen receptor α expression. The altered estrogen receptor α signaling decreased aromatase expression and estradiol production. Unfortunately, the question how 17β-HSD3 is induced by preeclampsia remains open. 17β-HSD1 also appears dysregulated – albeit significantly reduced – in preeclamptic placentas, most likely due to upregulation of miR-210 and miR-518c, which were confirmed to target 17β-HSD1 (Ishibashi et al., 2012). Dysregulation of miRNAs in preeclamptic placentas has also been reported by Hu et al. (2009) and Zhu et al. (2009).

The adrenocortical carcinoma cell line NCI-H295R is an established cellular model to study adverse effects on steroidogenesis of numerous substances including heavy metals such as cadmium (Knazicka et al., 2015) or industry-derived environmental toxins such as perfluoroalkyl acids (Kang et al., 2016). Various substances have already been found to change steroid production and thus it might be expected that these molecules could also impact placental steroidogenesis. For cadmium, this has already been demonstrated (Kawai et al., 2002), but further functional studies are required. In this context the recently established co-culture models of choriocarcinoma cells (JEG-3 or BeWo) with H295R cells should be considered for future analysis. The H295R/BeWo co-culture model offers the opportunity to evaluate the effects of chemical exposures on androgen and estrogen biosynthesis, as well as on various other aspects of feto–placental communication (Hudon Thibeault et al., 2017; Drwal et al., 2018; Thibeault et al., 2018).

### Glucocorticoids and the Placenta

**FIGURE 7:**
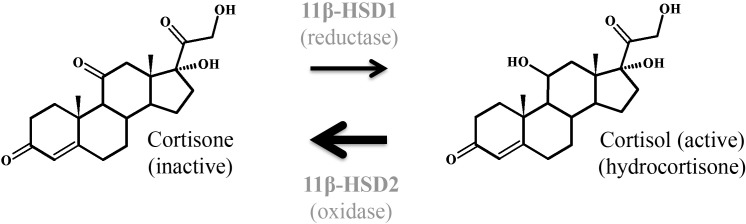
Interconversion of cortisol and the inactive metabolite cortisone by 11β-hydroxysteroid dehydrogenases type 1 and 2. 11β-HSD1 exerts mainly reductase activity *in vivo*, while 11β-HSD2 metabolizes the conversion of cortisol to cortisone. In human placenta, conversion from cortisol to cortisone predominates at all gestational ages, but increasing conversion of cortisone to cortisol in homogenized human placental tissue toward term indicates a predominant reductase activity of 11β-HSD1.

**FIGURE 8:**
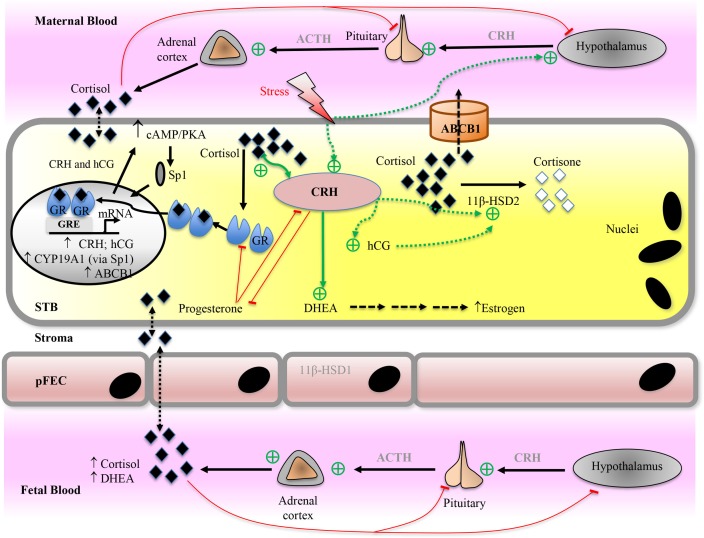
Proposed model for placental glucocorticoid (cortisol) function and metabolism. For detailed description, see text. Cortisol can diffuse across cell membranes and regulate target protein expression directly via glucocorticoid receptor (GR) or indirectly via other transcription factors (e.g., Sp1). Placental corticotropin-releasing hormone (CRH) is the major mediator of adaptive response to stressors during pregnancy. Cortisol stimulates placental CRH expression, which regulates placental hormone levels (e.g., hCG, estrogen, progesterone, and 11β-HSD2). Red arrows indicate inhibiting/negative feedback pathways, while green arrows indicate stimulating pathways. ABC transporters, ATP-binding cassette transporters; ACTH, adrenocorticotropic hormone; CRH, corticotropin-releasing hormone; DHEA, dehydroepiandrosterone; GRE, glucocorticoid response element; HSD, hydroxysteroid dehydrogenase; hCG, human chorionic gonadotropin; pFEC, placental-fetal endothelial cell; PKA, protein kinase A; Sp1, specificity protein 1 transcription factor; STB, syncytiotrophoblast.

Glucocorticoids or glucocorticosteroids are produced by the adrenal cortex in response to cues such as stress or illness under the control of the HPA-axis. The name glucocorticoid is derived from their ability to promote gluconeogenesis in the liver and their synthesis in the adrenal cortex (Castro et al., 2011). The natural human glucocorticoid is cortisol (**Figure 7**). In the adult, these signals stimulate the hypothalamus to release CRH, which in turn cause the release of ACTH from the anterior pituitary. ACTH induces synthesis of glucocorticoids from cholesterol in the adrenal glands, which exerts a negative feedback on the release of CRH and ACTH (**Figure 8**).

Glucocorticoids coordinate many functions such as inflammatory and immune responses, metabolic homeostasis, cognitive function, reproduction, and development. At the cellular level, glucocorticoids exert their effects by binding to the GR that is almost ubiquitously expressed and induces target gene transcription. The classical model of GR transactivation involves GR dimerization and binding at glucocorticoid response elements (GREs) leading to co-activator recruitment and activation of transcription from proximate promoters (Pavek and Smutny, 2014; Whirledge and DeFranco, 2018). GR is expressed in STB and CTB as well as in JEG-3 and BeWo cell lines (Pavek and Smutny, 2014). Cortisol stimulates productions of placental hormones such as CRH and hCG (Robinson et al., 1988; Ni et al., 2009). Increased cortisol levels in the placenta are linked to the induction of estrogen synthesis, which precedes the onset of parturition in human. Induction of CYP19A1 in trophoblast cultures in response to cortisol was shown to occur via activation of the cAMP/PKA pathway by CRH and hCG and the subsequent induction of transcription factor Sp1 (Wang et al., 2012, 2014; **Figure 8**).

#### Placental CRH Synthesis and Function

CRH is the major mediator of adaptive response to stressors and is synthesized by several organs (Koutmani et al., 2013; Slominski et al., 2013). During pregnancy, the CRH concentration in maternal plasma increases substantially and reaches levels that are 1,000–10,000 times that of non-pregnant women. The major CRH source during pregnancy is the placenta that can also produce ACTH (Bicknell, 2008; Gangestad et al., 2012; Thomson, 2013). CRH is synthesized in the STB from first to third trimester (Riley et al., 1991; Warren and Silverman, 1995). In humans and great apes CRH levels rise exponentially throughout pregnancy to peak at labor. Rodents, in contrast, do not exhibit placental CRH production (Heussner et al., 2016). Placental CRH production may have evolved in primates to stimulate fetal ACTH release and adrenal steroidogenesis, in order to guarantee sufficient synthesis of DHEA, a precursor for placental sex hormone synthesis. Concomitant stimulation of fetal cortisol and DHEA by placental CRH would couple the glucocorticoid effects on fetal organ maturation with the timing of parturition. While glucocorticoids inhibit hypothalamic CRH synthesis and secretion (Frim et al., 1990), they paradoxically stimulate placental CRH expression (Robinson et al., 1988; Jones et al., 1989; Wang et al., 2014).

CRH operates via activation of two receptors, CRH-receptor type 1 and type 2 (Grammatopoulos and Ourailidou, 2017), which are expressed in the human placenta (Florio et al., 2000). Placental CRH exhibits many functions in pregnancy and parturition. To name a few, CRH modulates placental glucose transporter expression (Gao et al., 2012) and stimulates estradiol production by induction of STS, CYP19A1, and 17β-HSD1 expression in trophoblasts (You et al., 2006). CRH impacts on the expression levels of several other placental hormones including ACTH (Challis et al., 1995) and prostaglandin (Gao et al., 2008). Progesterone is an inhibitor of CRH production (Karalis et al., 1996; Ni et al., 2004; Sfakianaki and Norwitz, 2006) and also a competitive antagonist of cortisol binding to GR (Majzoub and Karalis, 1999). On the other hand, CRH inhibits progesterone production by suppression of CYP11A1 and 3β-HSD1 (Jeschke et al., 2005; Yang et al., 2006). CRH is involved in the timing of birth by regulation of estrogen and progesterone levels as they control the contractile properties of the myometrium (Majzoub and Karalis, 1999; Gangestad et al., 2012; Thomson, 2013).

#### Placental Cortisol Metabolism and Transport in Health and Disease

Glucocorticoids are important during pregnancy and for fetal development. Fetal glucocorticoid synthesis is only partially influenced by the HPA axis, but instead is primarily regulated by differential expression of the enzymes required for glucocorticoid synthesis. Moreover, maternal glucocorticoids can potentially cross the placenta. To enable pregnancy and ensure proper fetal development, glucocorticoid signaling occurs during three period of gestation: early in pregnancy to enable implantation, between week 7 and 14 to enable fetal-adrenal development, repress DHEA synthesis and enable female genital development and finally during the third trimester. Fetal serum glucocorticoid levels must increase significantly before birth in order to ensure proper development of the lungs and several other organs (Busada and Cidlowski, 2017). On the other hand, the fetus should not be exposed to excessive levels of glucocorticoids; this can suppress fetal growth and program the fetus for life-long diseases such as hypertension, glucose intolerance, diabetes, and strokes (Moisiadis and Matthews, 2014a,b; Konstantakou et al., 2017).

In the adrenal gland, cortisol synthesis is initiated by 21-hydroxylase (CYP21A2) (Hanukoglu, 1992). CYP21A2 converts progesterone as well as 17α-OH-progesterone, through a hydroxylation at position C21, into 11-deoxycorticosterone and 11-deoxycortisol, respectively. After catalyzation by CYP21A2, 11β-hydroxylase (CYP11B1), and 11β-HSD are the key molecules mediating and regulating tissue-specific glucocorticoid actions. CYP11B1 catalyzes 11-deoxycorticosterone and 11-deoxycortisol to corticosterone and cortisol, respectively. There has been no evidence so far of CYP21A2 or CYP11B1 expression in human placenta. However, 11β-HSD type 1 and 2 isozymes are expressed in the placenta and the fetal membranes (Heussner et al., 2016; Yang et al., 2016; Konstantakou et al., 2017). 11β-HSD2 is an oxidase converting cortisol to the inactive 11-keto metabolite, cortisone. 11β-HSD1, in contrast, preferentially acts as a reductase *in vivo*, mediating the NADPH-dependent conversion of cortisone to cortisol (**Figure 7**). In line with this, Giannopoulos et al. (1982) reported increasing conversion of cortisone to cortisol in homogenized human placental tissue toward term, although conversion from cortisol to inactive cortisone predominated at all gestational ages.

Cortisol is required during early pregnancy for the establishment of gestation (Michael and Papageorghiou, 2008). 11β-HSD1 and GR are localized widely in the decidual stroma and epithelium, while the distribution of 11β-HSD2 is mainly confined to the decidual epithelium and scarcely observed in the decidual stroma. Furthermore, 11β-HSD1 is localized only in the fetal blood vessels in the interstitial core of the villous tissue but not in the extravillous trophoblast, CTB and STB. 11β-HSD2 expression is mainly restricted to the STB. The distribution pattern of 11β-HSD1 suggests that higher concentrations of cortisol are required on the maternal side than on the fetal side in early pregnancy (Yang et al., 2016).

Cortisol levels in the maternal circulation rise toward term (Goldkrand et al., 1976). As steroid hormones use free diffusion to enter target cells, maternal cortisol reaches placental cells. Overexposure of the fetus to glucocorticoids during pregnancy reduces birth weight and can be detrimental to fetal development. In the human placenta, 11β-HSD2 acts as a major “barrier” to materno-fetal cortisol transfer as shown in the isolated perfused placenta (Stirrat et al., 2018). 11β-HSD2 is localized abundantly in the STB (Yang et al., 2016) and generates a cortisone-to-cortisol-ratio >1 (Heussner et al., 2016). CRH and cortisol induce the expression of 11β-HSD2 in isolated trophoblasts (van Beek et al., 2004; Fahlbusch et al., 2012). Furthermore, cortisol stimulates hCG production in trophoblasts (Wang et al., 2014) and the upregulation of 11β-HSD2 expression in trophoblasts by cortisol may be mediated in part by hCG (Ni et al., 2009). Nevertheless, the conversion of cortisol is incomplete and a fraction of cortisol remains unmetabolized (Sun et al., 1999). The energy-dependent drug-efflux pump ABCB1 may mediate export of glucocorticoids from cells (Uhr et al., 2002). Studies in BeWo cells suggested that this transporter could contribute to the placental glucocorticoid barrier (Mark and Waddell, 2006). ABCB1 is expressed at the apical surface of the STB (Ni and Mao, 2011). In CTBs, the glucocorticoid drugs dexamethasone and betamethasone, significantly induce the expression of ABCB1 (Manceau et al., 2012). 11β-HSD2 and ABCB1 may thus act together to reduce fetal and placental exposure to maternal cortisol and thereby minimize the growth inhibitory action on the fetus (**Figure 8**).

11β-HSD1 and 2 are key molecules in the production and metabolism of glucocorticoids. Both are expressed in the human decidua and placenta and both are related to a number of pregnancy-associated complications. 11β-HSD1 is implicated in the pathogenesis of metabolic syndrome. 11β-HSD1 expression is altered in preeclampsia as well as IUGR and gene polymorphisms are associated with hypertensive disorders of pregnancy. Likewise, reduced 11β-HSD2 activity is related to preeclampsia, IUGR, and adverse pregnancy outcome (preterm birth). These interesting studies have been extensively reviewed in a recent publication (Konstantakou et al., 2017). Many of the underlying mechanisms causing altered expression of the enzymes remain to be explored, and additionally suspected correlations between altered enzyme expression and diseases such as GDM need to be confirmed. In addition, it remains to be demonstrated whether gene polymorphisms in 11β-HSD2 could serve as biomarkers for hypertensive disorders of pregnancy.

## Summary And Outlook

Cholesterol, progesterone, estrogens, and cortisol are required to establish and maintain pregnancy and ensure healthy fetal development. The human placenta, located at the interface of maternal and fetal circulation, has an active role in biosynthesis, metabolism, and transport of these molecules. Many enzymes and transporters are involved in these processes but our knowledge concerning their function and regulation is incomplete. The placental barrier is composed of trophoblast cells and pFECs. Few studies have addressed the role of pFECs in placental steroid handling. The functional interdependence of trophoblasts, pFECs, and fetal adrenal cells is incompletely understood. The use of co-culture systems may significantly broaden our understanding.

Diseases, but also external factors such as high fat diet or smoking alter the placental steroid metabolism. We need to explore these alterations and their potential consequences for fetus or mother. It should be kept in mind that the enzymes and transporters involved are regulated at multiple levels and by many endogenous molecules. Thus, whenever possible, mRNA levels, protein levels and posttranslational modifications should be examined (Hudon Thibeault et al., 2018). Likewise, when looking for changes in the concentration levels of steroids or other substances, subcellular fractionation should be considered in order not to miss important details (Lassance et al., 2015). Apart from diseases, we are facing an ever-growing number of toxic substances in the environment. As the steroid metabolism of the human placenta is crucial for life long health of fetus and mother, we should be interested to understand their influence on the function of the human placenta.

## Author Contributions

WC, KJ, and IE designed and wrote the article and met all criteria for authorship.

## Conflict of Interest Statement

The authors declare that the research was conducted in the absence of any commercial or financial relationships that could be construed as a potential conflict of interest.

## Abbreviations

7-DHC: 7-dehydrocholesterol
ABC: ATP-binding cassette
ACAT: Acyl-coenzyme A:cholesterol acyltransferase
ACTH: adrenocorticotropic hormone
A-dione: androstenedione
Apo: Apolipoprotein
ART: assisted reproductive technologies
BCRP: breast cancer resistance protein
CEH: cholesteryl ester hydrolase
CRH: corticotropin-releasing hormone
CTB: cytotrophoblasts
CYP: cytochrome P450
DHCR7: 7-dehydrocholesterol reductase
DHEA: dehydroepiandrosterone
DHEA-S: dehydroepiandrosterone sulfate
E1: estrone
E2: estradiol
E3: estriol
ER: endoplasmic reticulum
GDM: gestational diabetes mellitus
GR: glucocorticoid receptor
hCG: human chorionic gonadotropin
HDL: high-density lipoprotein
HMG-CoA: 3-hydroxy-3-methylglutaryl-coenzyme A
HMGR: HMG-CoA reductase
HPA axis: hypothalamic–pituitary–adrenal axis
HPEC: (isolated) human placental epithelial cells
HSD: hydroxysteroid dehydrogenase
HSP: heat shock protein
HUVEC: human umbilical vein endothelial cells
INSIG: insulin induced gene
IUGR: intrauterine growth retardation
LDL: low-density lipoprotein
LDLR: LDL receptor
LRP: LDLR-related protein
LXR: liver X receptor
MTP: microsomal triglyceride transfer protein
NPC: Niemann-Pick C
OAT: organic anion transporter
OATP: organic anion transporting polypeptide
OHC: hydroxycholesterol
OHP: hydroxyprogesterone
ORP: OSB-related protein
OSBP: oxysterol binding proteins
oxLDL: oxidized LDL
PCOS: polycystic ovary syndrome
PDI: protein disulfide-isomerase
pFEC: placental-fetal endothelial cells
PKA: protein kinase A
PM: particulate matter
RCT: reverse cholesterol transport
ROR: RAR-related orphan receptors
ROS: reactive oxygen species
RXR: retinoid X receptor
-S: -sulfate
SCAP: SREBP cleavage-activating protein
SCP: sterol carrier protein
SF: steroidogenic factor
sFlt1: soluble fms-like tyrosine kinase 1
SLC: solute carrier
SLCO, SLC transporter, SOAT: sodium-dependent organic anion transporter
SLOS: Smith-Lemli-Opitz syndrome
Sp1: Specific protein 1
SRB1: scavenger receptor class B type I
SRE: sterol regulatory element
SREBP: SRE-binding protein
StAR/STARD1: steroidogenic acute regulatory protein
START: StAR-related lipid transfer
STB: syncytiotrophoblast
STS: steroid sulfatase
SULT: sulfotransferase
TLR: Toll-like receptor
VLDL: very low-density lipoprotein
VLDLR: VLDL receptor
